# Digital stress and friendship conflict in adolescence: the role of perceived norms and features of social media

**DOI:** 10.3389/fdgth.2025.1497222

**Published:** 2025-03-18

**Authors:** Federica Angelini, Gianluca Gini

**Affiliations:** Department of Developmental Psychology and Socialization, University of Padua, Padua, Italy

**Keywords:** friendship conflict, digital stress, perceived norms, availability, social media features

## Abstract

**Introduction:**

Digital stress, resulting from expectations of online availability, can increase the risk of conflicts with friends. However, friendship conflict remains an underexplored indicator, particularly in association with stressful online experiences. This study aims to examine the association between digital stress and conflict levels overtime, considering the role of social media expectations.

**Method:**

1185 adolescents (59.3% f, Mage = 15.97 years, *SD* = 1.43) completed self-report measures at two timepoints, six months apart. A Structural Equation Model was employed to examine the longitudinal associations of social media expectations (i.e., friends' social media norms, friends' social media use, and perceived social media features) on friendship conflict, focusing on the mediating role of digital stress (i.e., entrapment and disappointment). Gender differences were explored.

**Results:**

Perceived norms about social media use and unique features of social media (i.e., visualness) contributed to explain digital stress and, in turn, friendship conflict. Specifically, emotional responses to unmet expectations of availability on social media (i.e., disappointment) emerged as particularly relevant in explaining conflictual interactions, compared to the perceived pressure to be responsive to friends (i.e., entrapment). Males perceiving high availability of social media experience lower levels of entrapment, compared to females.

**Discussion:**

Results from this study support the importance of considering social media as a context where to study friendship dynamics, as this knowledge can have several implications for promoting positive online experiences and preventing conflicts with friends.

## Introduction

**Figure 1:**
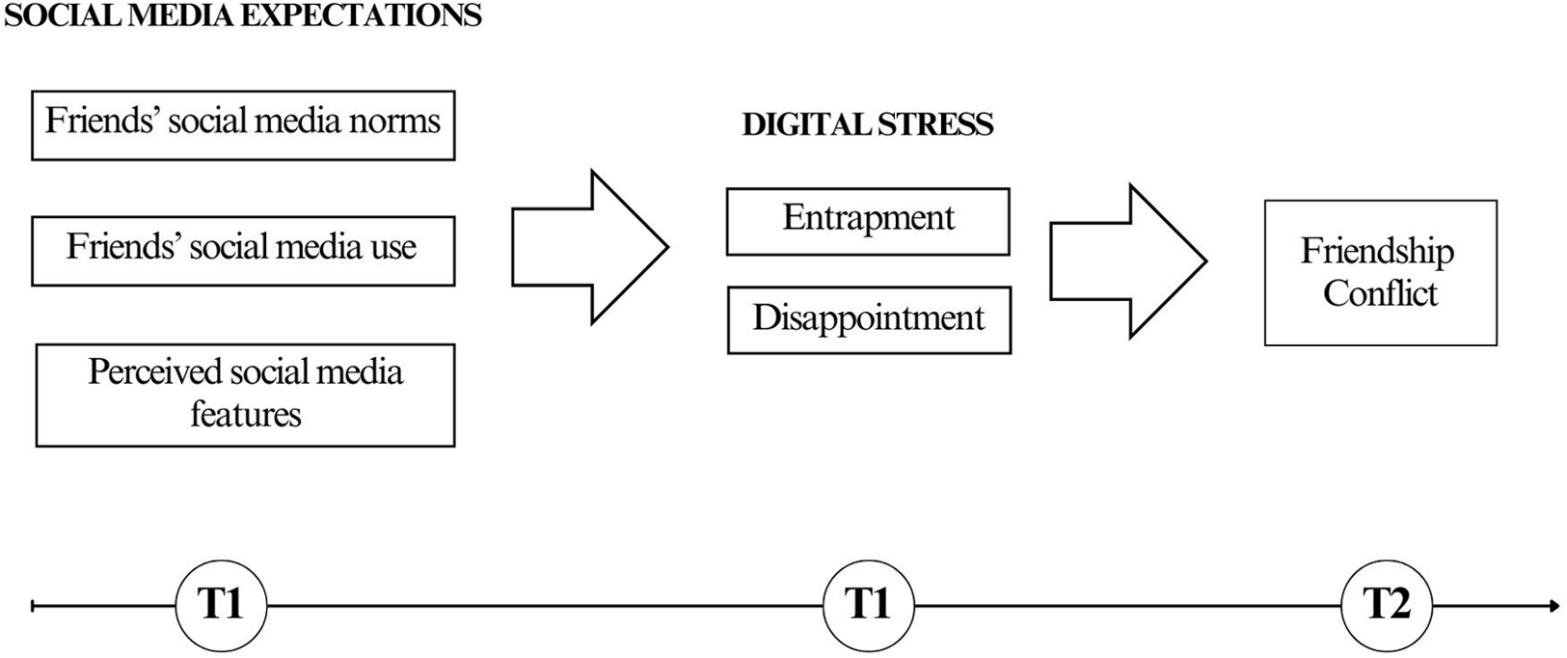
Hypothesized model.

Digital stress is a recent construct that captures different aspects of stressful experiences on social media related to expectation of availability, which can increase the likelihood of having conflicts with friends (1). Indeed, the reciprocal nature of friendship is reflected in adolescents' tendency to comply with their friends' expectations of availability, on the one hand, and the expectation that friends will behave in the same way, on the other. As friends’ interactions on social media have become a common aspect of contemporary relations (2–6), potential negative consequences may affect adolescents more than other age targets (7), especially within meaningful relationships such as friendships (1). Although the literature on digital stress and its association with various indicators of psychological wellbeing is consistent (see below), conflictual interactions between friends in association with social media use and digital stress during adolescence have rarely been investigated. Even fewer studies have analyzed this issue longitudinally. Indeed, the most recent findings about the negative impact of social media use come mainly from cross-sectional (1, 2) or qualitative [e.g., (8)] studies. Conversely, Nick et al. (9) recently found a longitudinal association between adolescents' digital stress and depressive symptoms. However, none of these studies specifically focused on friendship conflict, nor tested its longitudinal link with digital stress in a sample of adolescents. In addition, at least to our knowledge, perceived norms and features of social media have never been considered together as possible predictors of digital stress. Therefore, to contribute filling these gaps, in the current study, we examined social media expectations in terms of (i) friends' social media norms (i.e., participants' perceived importance to use social media for their friends), (ii) friends' social media use (i.e., participants perceived use of social media by their friends), and (iii) participants' perceptions of specific features of social media (i.e., availability, asynchronicity, cue absence, and visualness). Moreover, we investigated the experience of digital stress, conceptualized in two different ways, namely (i) entrapment (i.e., adolescents' perceived pressure to be constantly present for friends), and (ii) disappointment (i.e., negative feelings felt by adolescents' when friends are found to be not available to them). Finally, we examined the frequency of conflict with friends. Specifically, we longitudinally tested whether levels of conflicts between friends in the spring (T2) would be explained by social media expectations and digital stress assessed in the fall (T1) among a sample of adolescents. For sake of clarity, the conceptual model tested in this study is depicted in Figure 1. Furthermore, differences across gender groups were explored.

### Social media expectations

In the context of close relationships, friends develop expectations regarding how they ought to behave (3, 10). For example, expectation of reciprocal availability, whether in person (11, 12) or on social media, is a crucial aspect of relational maintenance during adolescence (4, 6, 13, 14). However, little research has investigated how adolescents' expectations about their friendships are developed in the context of social media. Therefore, as anticipated, in this study we focused on three contextual factors that may contribute to form social media expectations, that is, friends' social media norms, friends' social media use, and perceived social media features.

Specifically, friends' social media norms and friends' social media use can be framed within the literature about peer influence processes (15). Friendship, indeed, as a proximal social context, can have a strong effect in impacting adolescents' behavior and attitudes through various mechanisms such as conforming to peer group norms, shared interests and values, and social identity (16–18). Furthermore, it is well-known that adolescents tend to participate in activities deemed important by their peers, thus enhancing their sense of belonging to the group [Social Influence Theory; (19)]. In this regard, different types of social norms exist [e.g., (20, 21)] and can play a role, also on social media (22). In the current study, the extent to which using social media is perceived as a valuable and expected behavior by friends, that is *friends' social media norms*, can be referred to as an injunctive norm; this kind of norm, indeed, describes shared perceptions and expectations about what is valuable or desirable, or disapproved, and perceptions of how individuals feel that other group members ought to behave [e.g., “My friends think that I should spend a lot of time on social media”; (23)]. On the other hand, adolescents' perception of the frequency with which their friends use social media, that is, *friends' social media use* [especially by responding, liking, or commenting on participants' messages and content posted, that is, “other oriented social media use”; see (24)], is conceived as a descriptive norm, which refers to what group members do or the behaviors they frequently engage in (e.g., “My friends share photos or videos on social media depicting them and me together”). Whereas descriptive norms specify what is typically done among peers and are influential as they indicate what is sensible to do, the influence of injunctive norms stems from individuals' motivation for affiliation, as they indicate what should be done and are, therefore, more related to potential social sanctions (20, 21). However, descriptive and injunctive norms are often congruent in the sense that what most people do in a given social context is often what one is expected to do, and both concepts have in common the pressure that individuals experience to conform with the group (25, 26). Although previous studies have primarily focused on the role of perceived social norms in the development of online risky behavior, such as problematic use of video games [e.g., (27, 28)] and of social media; [e.g., (23)], it has been shown that higher perceived importance and higher engagement in online activities by friends can shape a variety of adolescents' behavior in the digital environment. For example, in a study with a sample of Italian adolescents (27), it was found that perceiving that friends use social media frequently (i.e., friends' social media use) increased participants' engagement in social media activities to interact with them and created the expectation that friends would behave similarly. Specifically, friends' (other-oriented) social media use embeds the implicit intention to interact with others, that is why it is reasonably associated with the pressure to interact with, or conversely, with the expectation to receive responses from friends. Thus, when conceptualized as such normative processes (i.e., what is most often done or approved by friends), both friends' social media norms and friends' social media use may contribute to form the expectation that friends will use social media, which may result in stressful online experiences. Indeed, individual expectations can vary, and misunderstandings and negative feelings can arise when friends have different assumptions about each other's online availability. In this regard, recent studies have discussed the role of digital social norms in users’ well-being, by distinguishing between descriptive and injunctive norms [e.g., (22)], and between intrinsic and extrinsic motives of social media use [e.g., (29, 30)]. For example, it has been shown that being more present in face-to-face interactions to meet social norms of a healthier use of social media (i.e., digital disconnection) contradicts the expectations of availability toward online interactions (22); in particular, although digital disconnection is perceived more as what should be done (injunctive norm), it has to deal with the pressure to be available, so that, for younger individuals, remaining available online is perceived as less harmful than being disconnected (22). In other words, the desire of autonomy clashes with the need to fulfil others' expectations (31, 32). In addition, beyond intrinsic motivations to use social media (e.g., need satisfaction), which are usually associated to positive feelings, extrinsic motivations to comply with availability norm are associated with lower self-control behaviors and related negative emotions (29). For example, users may feel guilty when they do not check messages or do not reply promptly [e.g., (29)]. Therefore, while social media have facilitated connection and communication, which can benefit online social support processes and emotional expression with friends (2), they have also given rise to a cycle of expected availability among friends (7, 8), which represents a new relational stressor for adolescents' experiences with their friends on social media. Hall & Baym (33) have looked at a similar concept, describing how expectations of relational maintenance with friends through mobile phone (i.e., mobile maintenance expectations) may have contradictory effects, allowing for a greater sense of inclusion but also to feelings of imprisonment due to constant exchanges with friends.

Beyond friends' social media norms and friends' social media use, another “contextual” dimension that may contribute to explaining adolescents' social media expectations within friendship refers to unique features of social media (i.e., *perceived social media features*). Baym (34) has described seven key concepts that characterize media communication to varying degrees (i.e., interactivity, asynchronicity, social cues, storage, replicability, reach and mobility), and that can give rise to new possibilities and behavior compared to face-to-face interactions. Mobility, in particular, refers to the extent to which media are portable, thus allowing interactions to be made possible regardless physical location, but also threatening individuals' autonomy. With regard to a similar, current, theoretical framework that describes social media as a relational context, Nesi et al. (3–5) have recently introduced the Transformation Framework, according to which specific features (i.e., asynchronicity, permanence, availability, publicness, cue absence, quantifiability, visualness, and algorithm), contribute to transform the way adolescents, especially friends, interact with each other. Specifically, in line with the Transformation Framework, the perceived presence of unique features of social media can make some online behavior more or less appropriate and likely. In other words, adolescents' perception of social media context, with its own features, may have altered relational dynamics and created new expectations and demands within meaningful relationships (8, 35), such as expectations for accessibility, feedback-seeking or online multitasking behaviors (13, 36). Among the eight social media features identified within the Transformation Framework, some more than others may encourage a high frequency of contact with friends or create relational uncertainty (13, 37). For example, the feature of *availability* allows for accessibility to friends anytime and from anyplace; while this may allow one to stay in touch with distant friends or to easily share content regardless of the physical location, it may also create expectations about reciprocal, constant access to interactions and social support, and lead to too many communication demands [i.e., communication overload; (32, 38)]. Furthermore, the possibility to have asynchronous conversations on social media (i.e., *asynchronicity*) allows for more comfortable, even multiple interactions with friends, thank to time intervals between the aspects of communication, which may also occur simultaneously with other online activities [i.e., media multitasking; (39)]. However, not responding promptly enough to a friend's message, or not receiving a quick response by friends during a conversation, even when they are visibly online, may increase perceived pressure to be available, as well as relational uncertainty and higher reassurance seeking (22). Lack of social cues on social media, such as tone of voice or facial expressions (i.e., *cue absence*), can increase comfort and self-disclosure during online conversations, but it can also lead to misunderstandings due to misinterpretation of messages (37). Additionally, requests for social support may not be fulfilled due to the reduced perceived richness of interactions (2). Finally, the high visibility of content on social media through photos and videos (i.e., *visualness*) allows for novel opportunities of communication with friends; however, it can also create expectations that friends will comment on posted visual content [e.g., (40)], or make adolescents more easily aware of what friends are doing and of their availability (e.g., seeing from friends' social media profiles that they were having fun elsewhere after they said they could not hang out), consequently increasing feelings of jealousy, insecurity, rejection, or social comparison [e.g., (41)]. On the other hand, adolescents may feel inhibited to share new content showing where they are and what they are doing at that moment, to avoid letting their friends know that they are online (25). Therefore, social media and their functions, together with online peer influence processes, would contribute to adolescents' expectations (and compliance) of relationship maintenance through social media, which may be associated with negative experiences and feelings related to social media use among friends.

### Digital stress

The internalization of social media expectations favoured by friends' social media norms, friends' social media use and perceived social media features may contribute to increased pressure to conform to friends' demands, as well as to meet one's own needs. Within the social media environment, such pressure has been defined as “*digital stress*” (42); this broad term refers to cognitive, affective, and behavioral responses to what occurs on social media. Specifically, the subjective experience of digital stress is associated with individuals' ability to handle exceeding coping resources within their relational contexts (32, 43). Moreover, the construct of digital stress comprises other specific sub-terms (42, 44). In particular, the word “*entrapment*” has been used to identify users' sense of pressure resulting from the need to fulfil someone else's expectations of being available on social media, such as expectations of being promptly responsive to messages or new content (1, 33). Other studies have described similar concepts using different terms, such as “social pressure” [e.g., (45, 46)], “accessibility stress” (44, 47), or “availability stress” (42). To some extent, entrapment may also result from the feeling of guilt to be not available to friends or the fear of facing negative consequences or conflicts with friends. In this regard, other authors have explained the contradiction between individuals' expectations of closeness and the desire to be independent [e.g., (48, 49)]. For example, some adolescents may choose to reply immediately on social media with the goal of preventing arguments; however, prolonged compliance with friends' expectations and lack of care for one's own needs may even increase the pressure they want to avoid, thus leading to a negative outcome anyway.

Other dimensions of digital stress have received less attention. Specifically, a particular form of stress that may be defined as “social media disappointment” (henceforth “*disappointment*”) has not received enough attention in social media research. A similar concept, that is “social media stress”, has been introduced in previous research (50, 51). Here, disappointment refers to users' feelings of sadness, anger, or frustration when the expectations that friends will respond to their messages or react to new posts immediately after they have been sent or published on social media are not met (52). In other words, while entrapment is related to feeling guilty over the failure in meeting others' expectations, disappointment is associated with a feeling of anger at friends' failure to meet one's own expectations (29). Therefore, entrapment and disappointment represent the two sides of digital stress related to the expectation of being available and reflect one of the core aspects of friendship, that is, reciprocity within the relation. In addition, these two facets of digital stress may be interrelated because, as in a vicious circle, not receiving timely responses may lead some adolescents to push their friends to fulfil their requests. However, they are also expected to play a unique role in mediating between social media expectations and conflicts between friends.

Recent research, indeed, has begun to investigate the effects of the pressure to be available among young individuals, with a focus on negative general and mental health outcomes, such as increased stress, depression, anxiety [e.g., (44, 53)], and poorer sleep quality [e.g., (54, 55)]. Similarly, feelings of being ignored or excluded on social media (e.g., not getting a prompt response from friends after sending a message or posting new content) seem to negatively affect individuals' well-being and to increase feelings of rejection [e.g., (56, 57)], loneliness, and social anxiety [e.g., (9)]. In addition, the perceived pressure of availability seems to characterize especially close relationships and is associated with decreased relational satisfaction and poorer social connection among both adolescents (1, 7) and young adults (31, 42).

In this regard, digital stress represents a novel, additional stressor for adolescents, which could lead to an increased frequency of conflicts, thus undermining the perceived quality of their friendship (42).

### Friendship conflict

Friendship conflict is a common and natural part of the development of peer relationships during adolescence; in a period of significant changes, indeed, friendship can be both a source of support and a potential source of conflict (11, 12, 58). Arguments with friends may arise from various triggers, such as differences in values, beliefs, or interests, especially when adolescents are torn between their own needs, on the one hand, and the desire to conform to certain behaviors and norms within the peer groups, on the other. In this regard, a recent study by Fox et al. (1) found that adolescents' perceived pressure to be available to friends on social media (i.e., entrapment) increased friendship conflicts, especially among males. However, there is little, and mostly qualitative evidence about the association of digital stress with adolescents' negative experiences with friends [e.g., (8)], and even less about conflictual interactions. Therefore, this study aimed to contribute to expanding research in this field. Specifically, friends' social media norms [e.g., (23)] and friends’ social media use [e.g., (27)] will be analyzed together to investigate whether social media expectations may represent a potential source for the experience of digital stress and, in turn, for friendship conflict. In doing so, as a major novelty, friendship conflict will be investigated taking into account both aspects of digital stress, which have never been studied together so far. In our conceptualization of the model, the two mediators are considered temporally contiguous on social media expectations; the two dimensions of digital stress, indeed, represent possible cognitive and emotional reactions that are an immediate and almost automatic consequence of the processing of the relational context, that is, perceived norms and features of social media. In addition, since the focus of the study is on conflict predictors, we required a time interval between mediators and outcome. Therefore, we tested the potential mediating role of digital stress measured at T1.

### Gender differences

Regarding gender differences, it is well known that among female adolescents, on average, perceived group norms might include the expectation for greater emotional connection and validation in close friendships and to show stronger emotional responses to both in-person (12) and online social interactions, particularly in stressful situations [e.g., (6, 44)]. Furthermore, females also show higher engagement in social media activities and preference for online interaction compared to males, who tend to disclose their emotions less both online and offline and to rely more on offline interactions to fulfil their relational needs (36, 75). In a recent study with a sample of Italian adolescents (2) it was found that frequent online interactions with friends, facilitated by the accessibility of social media, were associated with greater perceived validation within friendship for females, while it reduced perceived validation among males. Similarly, in a study by Pouwels et al. (14), WhatsApp use was found to decrease perceived friendship closeness among males, compared to females. Furthermore, it was recently found that males experiencing entrapment on social media reported higher levels of conflict with friends over time (1).

However, research in this field is still very limited. Therefore, given the lack of existing studies explicitly focused on the specific variables considered in the current work, the analyses including gender were deemed exploratory, and we did not formulate any a-priori hypothesis about gender differences.

### The current study

Based on the above literature and previous findings, using a short-term longitudinal design, the current study aimed to explore the role of social media expectations—represented by friends' social media norms (i.e., injunctive norms), friends’ social media use (i.e., descriptive norms), and the perception of four social media features, namely asynchronicity, availability, cue absence, and visualness—in explaining the experience of digital stress on social media (i.e., entrapment and disappointment) and, in turn, friendship conflicts during adolescence (see Figure 1). In addition, we were interested in exploring possible gender differences (females vs. males) in the tested associations.

Specifically, according to the existing literature on peer influence processes during adolescence, we expected that a higher perception of injunctive and descriptive norms about using social media within the peer group would be associated with a higher perceived pressure to be available (entrapment) and with increased emotional distress when friends are not available to participants (disappointment):

H1. Friends' social media norms and friends' social media use are positively associated with both entrapment and disappointment.

Second, because failure to meet one's own and others' expectations of availability can threaten relational well-being, we expected both aspects of digital stress to mediate the associations of perceived norms with increased friendship conflict:

H2. Friends' social media norms and friends' social media use are indirectly and positively associated with friendship conflict via both experiences of entrapment and disappointment.

Third, we expected that a higher perception of the presence of specific social media features would be associated with a higher perceived entrapment and disappointment.

H3. Asynchronicity, availability, cue absence, and visualness are positively linked to both entrapment and disappointment.

Finally, we expected an increased feeling of digital stress to mediate the associations of perceived social media features and friendship conflict:

H4. Asynchronicity, availability, cue absence, and visualness are indirectly and positively linked to friendship conflict, via both experiences of entrapment and disappointment.

## Materials and methods

### Sample and procedure

A total of 1,334 students in grades 9th to 13th (typically aged 13–14 when entering grade 9th) attending public secondary schools in Italy participated in the study. All participants reported using at least one social media platform, which was the only inclusion criterion for this study, in addition to age. The first wave of data collection occurred between November and December 2022 (T1). A total of 1,185 students (59.3% females, mean age = 15.97 years, *SD* = 1.43) completed the study measures. A total of 1,096 students (52.8% females, mean age = 15.93 years, *SD* = 1.40) participated in the second wave, which occurred between April and May 2023 (T2). An anonymized alphanumeric code was used to match T1 and T2 data. Students who participated in both T1 and T2 were 947, while those present only at T1, or only at T2 were 238 and 149, respectively. Thus, the retention rate was 88.35%. Attrition analyses were performed to examine the differences between students who participated in both waves and those who were missing at T2. Findings indicated there were no significant mean differences in both conflict scores, disappointment, friends' social media use, perceived visualness, and cue absence. Differential attrition based on gender emerged (χ^2^ = 4.39, *p* = .036), showing that 61.1% of participants in both waves and 52.1% of those present only at T2 were females; furthermore, students who did not participate in the second wave were slightly older than those who took part in both waves (*M*_age_ = 16.14 vs. *M*_age_ = 15.93*; t* = 2.07, *p* = 0.019); they also reported lower levels of perceived asynchronicity (*M* = 3.48 vs. *M* = 3.67; *t* = −3.16, *p* < .001) and availability (*M* = 4.19 vs. *M* = 4.30; *t* = −1.93, *p* = 0.027), and higher perceived friends' social media norms (*M* = 1.96 vs. *M* = 1.84; *t* = 1.99, *p* = 0.023) and feeling of entrapment (*M* = 1.81 vs. *M* = 1.73; *t* = 1.65, *p* = 0.049).

The study protocol and procedure were approved by the local Ethics Committee for Research in Psychology of the University of Padova. First, authorization from school principals was obtained and parents or students aged 18 years or older signed active consent. Data collection occurred twice within one school year, during a regular school-day. A graduate research assistant was present during data collection; participants were informed that they could leave the study at any stage without consequences, and confidentiality was assured. At the end of data collection, any questions or doubts about the questionnaires were addressed and the participants were thanked for their time.

### Measures

#### Friendship conflict (T1 and T2)

The frequency of conflict with friends was assessed with two measures: (i) three items from the “Conflict” subscale of the Friendship Quality Questionnaire–Revised (FQQ; 12, 59) and (ii) the 7-item Partner-Specific Rejecting Behaviors Scale (60).

Regarding the conflict subscale of the FQQ, participants were asked to think about their closest friends and to rate items on a 5-point scale (from 1 = not at all, to 5 = completely true). A sample item is: “My friend and I argue a lot”. This measure has been used in previous studies with adolescents and has demonstrated good psychometric qualities [e.g., (2, 59, 61)]. In this sample, the scale confirmed a good longitudinal factorial structure (CFA: χ^2^ = 3.012, *p* = .884, CFI = 1, RMSEA = 0.000, SRMR = 0.007), with factor loadings between 0.427 and 0.722 (*p* < .001) at T1, and between 0.553 and 0.729 (*p* < .001) at T2. The internal consistency at T1 was Cronbach's *α* = 0.58 (95% CI = 0.54–0.62), McDonald's *ω* = 0.64, and Cronbach's *α* = 0.69 (95% CI = 0.66–0.72), McDonald's *ω* = 0.71 at T2.

The 7-item Partner-Specific Rejecting Behaviors Scale (60) was adapted to friendship by Mackinnon et al. (62). Examples of items are: “I get angry or irritated with my friends”, “I am impulsive or selfish with my friends”. Participants were asked to think about their closest friends and to rate items on a 5-point scale (from 1 = not at all, to 5 = completely true). This measure has been used in previous studies with adolescents and has demonstrated good psychometric qualities [e.g., (62, 63)]. In this sample, the scale confirmed an adequate longitudinal factorial structure (CFA: χ^2^ = 507.397, *p* < .001, CFI = .910, RMSEA = 0.070, SRMR = 0.051), with factor loadings between 0.491 and 0.628 (*p* < .001) at T1, and between 0.559 and 0.737 (*p* < .001) at T2. Internal consistency at T1 was Cronbach's *α* = 0.76 (95% CI = 0.73–0.78), McDonald's *ω* = 0.77, and Cronbach's *α* = 0.81 (95% CI = 0.79–0.83), McDonald's *ω* = 0.82 at T2. Answers to each item were averaged for each scale, then in the study model a latent variable for “friendship conflict” with these two measures was computed in both waves.

#### Friends' social media norms (T1)

To measure injunctive norms about social media use within the friends' group, we used a 4-item scale by Marino et al. (23). Examples of items are: “My friends think that I should spend a lot of time on social media”, “My friends think that it is important that I use social media a lot”. Participants responded to each item on a 5-point scale (from 1 = not at all, to 5 = completely true). This measure has been used in previous studies and has demonstrated good psychometric qualities [e.g., (23, 64, 65)]. In this sample, the scale confirmed a good longitudinal factorial structure (CFA: χ^2^ = 182.733, *p* < .001, CFI = 0.958, RMSEA = 0.086, SRMR = 0.059), with factor loadings between 0.520 and 0.862 (*p* < .001). The internal consistency was Cronbach's *α* = 0.79 (95% CI = 0.77–0.81), McDonald's *ω* = 0.77. A mean score was computed with the 4 items.

#### Friends' social media use (T1)

To assess descriptive norms about perceived frequency of friends' use of social media, we used three items that have been created *ad hoc* in a previous study (27), because no measures were available. The three items are the following: “My friends react to my posts/stories with likes or comments”, “My friends tag me in a post/story”, and “My friends share photos or videos on social media depicting them and me together”. Participants rated each item on a 5-point scale (from 1 = never, to 5 = very often). This scale has already demonstrated good psychometric structure (27). In this sample, the scale confirmed a good longitudinal factorial structure (CFA: χ^2^ = 31.705, *p* < .001, CFI = 0.992, RMSEA = 0.057, SRMR = 0.018), with factor loadings between.644 and.839 (*p* < .001). The internal consistency at T1 was Cronbach's *α* = 0.82 (95% CI = 0.82–0.84), McDonald's *ω* = 0.83. A mean score was computed with the 3 items.

#### Perceived social media features (T1)

To assess adolescents' perceived presence of specific social media features, we used the Perceived Social Media Features Scale (2). This scale was developed to address the lack of a proper measure that captures adolescents' actual perceptions of how social media platforms are structured and operate. Moreover, this measure enables the differentiation of the distinct impact of each feature, beyond focusing on specific platforms. The whole scale includes a total of 16 items that were developed starting from the conceptual definition of each social media feature (5, 13). For the purpose of the current study we measured Asynchronicity (2 items: “I think that during interactions with others on social media it is possible to take time before answering”, “I think that during interactions with others on social media it may be some time before receiving an answer”), Availability (2 items: “I think it is easy to share new content on social media or to access content shared by others regardless of where we are (e.g., even when we are distant from those people)”, “I think it is easy to contact other people and communicate with them through social media, even when we are distant”), Cue absence (2 items: “I think that during social media interactions some aspects of communication (such as voice tone, gestures and facial expressions) may be absent”, “I think that social media interactions may lack some communicative signals such as voice tone, gestures and facial expressions”), and Visualness (2 items: “I think that on social media, photos and videos allow you to express yourself”, “I think that on social media communication through photos and videos is very important”). Participants rated their level of agreement with each item on a 5-point scale (from 1 = not at all, to 5 = completely true). The scale has already been used with Italian adolescents and has shown good factorial validity (2, 64). In this sample, the scale confirmed a good longitudinal factorial structure (CFA: χ^2^ = 234.321, *p* < .001, CFI = 0.968, RMSEA = 0.040, SRMR = 0.025), with factor loadings between 0.562 and 0.823 (*p* < .001). The internal consistency was McDonald's *ω* = 0.63 for asynchronicity, 0.65 for availability, 0.80 for cue absence and 0.54 for visualness. For each feature, a mean score was computed.

#### Entrapment on social media (T1)

To assess the perceived pressure to be available on social media for friends, we used items from the Entrapment and Maintenance of Expectations Scale (EMES) by Hall and Baym (33). The scale includes 5 items (e.g., “I feel pressured that I have to be available to my friends through social media”, “I feel pressured to use social media to keep in touch with my friends”). Participants responded on a 5-point scale (from 1 = not at all, to 5 = completely true). This scale has been recently used with adolescents showing good psychometric qualities [e.g., (1, 41)]. In this sample, the scale confirmed a good longitudinal factorial structure (CFA: χ^2^ = 333.636, *p* < .001, CFI = 0.901, RMSEA = 0.081, SRMR = 0.047), with factor loadings between 0.479 and 0.710 (*p* < .001). The internal consistency was Cronbach's *α* = 0.75 (95% CI = 0–73–0.77), McDonald's *ω* = 0.76.

#### Disappointment on social media (T1)

To measure feelings of sadness and upset after not being considered on social media, we used items adapted from the emotional responses subscale of the Social Media Stress scale by van der Schuur et al. (51). The original subscale included 5 items, most of which focused on social media dependency (e.g., “I feel tensed or restless when I can not use social media”); thus, for the purpose of the current study only 2 items were used after being slightly modified, and a third one was created *ad hoc*: “I feel disappointed when I do not get an immediate response if I have posted or sent something to my friends on social media”, “I feel disappointed when I do not receive any message or notification from my friends on social media”, “I feel disappointed when my friends are not available on social media”. This scale has been recently used with adolescents showing good psychometric qualities [e.g., (52)]. In this sample, the scale confirmed a good longitudinal factorial structure (CFA: χ^2^ = 84.095, *p* < .001, CFI = 0.970, RMSEA = 0.084, SRMR = 0.030), with factor loadings between 0.678 and 0.788 (*p* < .001). The internal consistency was Cronbach's *α* = 0.79 (95% CI = 0.76–0.81), McDonald's *ω* = 0.79.

### Data analyses

Analyses were performed using Mplus 8.3 (66); for applications see for example (67), with the maximum likelihood estimator. In the tested model, friendship conflict was the latent dependent variable assessed at T2. Social media expectations (i.e., injunctive and descriptive norms about social media use, and the four perceived social media features) were the independent variables assessed at T1. Adolescents' experiences of entrapment and disappointment on social media were included in the model as T1 mediators. Moreover, we controlled for participants' age and for baseline level of friendship conflict, and we included in the model the contemporaneous correlations between the independent variables measured at T2 with the dependent variable. Furthermore, since data were collected in school classrooms, we ran the model using the “type = complex” feature to account for dependency among observations (i.e., students nested within classes), by defining the classroom as cluster variable.

The model was first tested on the entire sample of adolescents, then a multi-group analysis was run to evaluate the model separately for both genders (males vs. females) and the null hypothesis of parameter equalities across groups was tested using the Wald chi-square test in Mplus (68–70, pp. 276–278). To evaluate the goodness of the model, *R^2^* was examined for each endogenous variable. Indirect effects were calculated using bias-corrected bootstrap confidence intervals, with 10,000 bootstrapped iterations, that were considered significant when zero was not included in the 95% confidence interval.

## Results

### Descriptive statistics and correlations

Descriptive statistics and correlations among the study variables in the whole sample and in the group of females vs. males are reported in Tables 1–3. As expected, both entrapment and disappointment were correlated with the two conflict scores both at T1 and T2, as well as with friends' norms about social media use in the whole sample and across gender groups; however, few correlations between social media features and digital stress emerged.

**Table 1:** Descriptive statistics of the study variables.

Variables	M (*SD*)		
	Whole sample	Females	Males
1. Conflict (T1)	2.05 (.62)	1.95 (.62)	2.21 (.59)
2. PSRB (T1)	1.71 (.55)	1.65 (.49)	1.81 (.59)
3. Conflict (T2)	2.17 (.70)	2.02 (.67)	2.33 (.63)
4. PSRB (T2)	1.86 (.63)	1.72 (.54)	2.02 (.67)
5. Friends’ SM norms (T1)	1.87 (.77)	1.85 (.75)	1.88 (.75)
6. Friends’ SM use (T1)	3.43 (1.07)	3.71 (.95)	3.02 (1.11)
7. Asynchronicity (T1)	3.63 (.83)	3.70 (.83)	3.47 (.79)
8. Availability (T1)	4.28 (.76)	4.36 (.70)	4.16 (.81)
9. Cue absence (T1)	3.97 (.99)	4.12 (.92)	3.74 (1.05)
10. Visualness (T1)	2.87 (.84)	2.92 (.84)	2.80 (.84)
11. Entrapment (T1)	1.75 (.70)	1.79 (.75)	1.67 (.63)
12. Disappointment (T1)	1.88 (.85)	1.95 (.88)	1.79 (.81)
13. Age (T1)	15.97 (1.43)	15.91 (1.52)	16.08 (1.25)

Notes: *N* = 1,334; *N* (females) = 703; *N* (males) = 469. PSRB, partner-specific rejecting behaviors; SM, social media.

**Table 2:** Correlations between the study variables in the whole sample.

Variables	1	2	3	4	5	6	7	8	9	10	11	12
1. Conflict (T1)	–											
2. PSRB (T1)	.40**	–										
3. Conflict (T2)	.51***	.33***	–									
4. PSRB (T2)	.29***	.53***	.50***	–								
5. Friends’ SM norms (T1)	.20***	.22***	.14***	.12***	–							
6. Friends’ SM use (T1)	−.03	−.03	.01	−.01	−.01	–						
7. Asynchronicity (T1)	−.04	.06*	−.08**	.02	−.01	.05*	–					
8. Availability (T1)	−.07*	−.02	−.08**	−.05	−.08**	.16***	.38***	–				
9. Cue absence (T1)	−.09***	−.06*	−.11***	−.05*	−.06*	.06*	.31***	.32***	–			
10. Visualness (T1)	−.01	.07**	.04	.03	.08**	.23***	.18***	.23***	.03	–		
11. Entrapment (T1)	.13***	.17***	.10***	.13***	.47***	.06*	.03	−.04	.01	.10***	–	
12. Disappointment (T1)	.11***	.25***	.16***	.21***	.38***	.09***	.04	.01	.01	.17***	.51***	–
13. Age (T1)	−.01	.12***	.02	.06*	.03	−.05*	.09***	.04	.12***	.02	.10***	.07*

Notes: *N* = 1,334. PSRB, partner-specific rejecting behaviors; SM, social media.

* *p* *<* .05.

** *p* *<* .01.

*** *p* < .001.

**Table 3:** Correlations between the study variables across gender groups (females: below the diagonal; males: above the diagonal).

Variables	1	2	3	4	5	6	7	8	9	10	11	12	13
1. Conflict (T1)	–	.36***	.48***	.26***	.25***	.04	−.01	.02	−.03	.07	.17***	.14***	.04
2. PSRB (T1)	.42***	–	.28***	.48***	.25***	.05	−.03	−.03	−.01	.03	.15***	.26***	.01
3. Conflict (T2)	.50***	.33***	–	.51***	.17***	.11*	−.04	−.03	−.10*	.07	.16***	.22***	−.06
4. PSRB (T2)	.25***	.55***	.42***	–	.21***	.12*	−.08	−.04	−.03	.01	.18***	. 29***	−.02
5.Friends’ SM norms (T1)	.17***	.20***	.13***	.07*	–	.01	−.05	−.12**	−.12**	.09*	.49***	. 44***	−.05
6. Friends’ SM use (T1)	.03	−.02	.09*	.01	−.01	–	.01	.13**	.06	.27***	.04	.04	.05
7. Asynchronicity (T1)	−.01	.19***	−.04	.17***	.01	−.01	–	.39***	.28***	.18***	−.12**	−.08*	.01
8. Availability (T1)	−.08*	.04	−.05	.01	−.05	.12***	.34***	–	.34***	.21***	−.19***	−.12**	−.06
9. Cue absence (T1)	−.08*	−.05	−.05	.01	−.01	−.05	.28***	.26***	–	.02	−.09*	−.05	.12**
10. Visualness (T1)	−.05	.13***	.04	.09*	.08*	.18***	.15***	.22***	.01	–	.05	.09*	.03
11. Entrapment (T1)	.15***	.20***	.10**	.14***	.46***	.02	.09**	.04	.04	.12***	–	.52***	.03
12. Disappointment (T1)	.13***	.28***	.16***	.21***	.35***	.08*	.07*	.07*	.01	.20***	.50***	–	.06
13. Age (T1)	−.01	.16***	.03	.08*	.07*	−.04	.15***	.11**	.14***	.05	.14***	.07*	–

Notes: *N* (females) = 703; *N* (males) = 469. PSRB, partner-specific rejecting behaviors; SM, social media.

* *p* *<* .05.

** *p* *<* .01.

*** *p* < .001.

### Analysis on the whole sample

**Figure 2:**
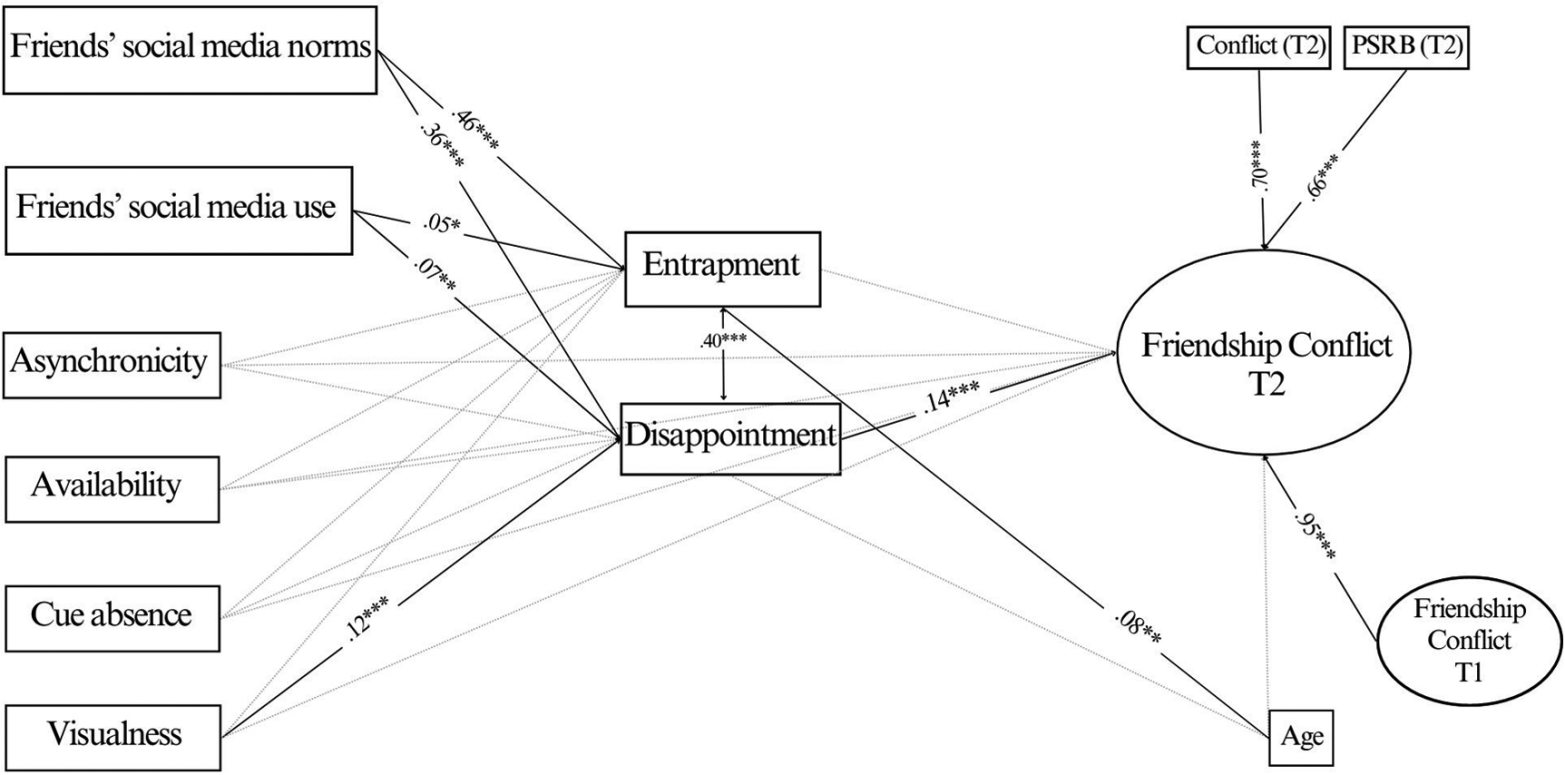
Model of the relationships between the study variables in the whole sample. Notes: *N* = 1,184; *p *<* .05, **p *<* .01, ***p *<* .001. For sake of clarity, only significant associations are reported.

First, we run the SEM model on the whole sample. Significant standardized results are reported in Figure 2. Regarding direct associations, we found significant positive associations of both friends' social media norms and friends' social media use with disappointment (*β* = 0.361, *p* < .001; *β* = 0.065, *p* = .007), and with entrapment (*β* = 0.462, *p* < .001; *β* = 0.052, *p* = .022). After controlling for baseline levels of friendship conflicts, only disappointment was found to positively predict friendship conflict (*β* = 0.144, *p* < .001). On the contrary, entrapment was not significantly associated with the outcome (*β* = −0.033, *p* = .495). Finally, among the four social media features, only visualness was found to be significantly and positively associated with disappointment (*β* = 0.124, *p* < .001). The control variable age was found to be weakly associated only with entrapment (*β* = 0.079, *p* = .010).

Overall, the model explained 92.8% of the variance for friendship conflict, and the explained variance for the mediators was 16.4% for disappointment and 23.6% for entrapment.

In addition to direct paths, three significant indirect effects emerged (Table 4). The strongest indirect association was found between friends' social media norms and friendship conflict via disappointment, and a smaller one, of borderline statistical significance, between friends' social media use and friendship conflict. Finally, we also found another mediation effect of disappointment in the association between visualness and conflict.

**Table 4:** Unstandardized indirect effects.

Independent variables	Mediators	Friendship conflict	
		ES	CI 95%
Friends’ SM norms (T1)	Entrapment	−0.008	−0.033–0.016
Friends’ SM norms (T1)	Disappointment	0.029	0.011–0.050
Friends’ SM use (T1)	Entrapment	−0.001	−0.004–0.001
Friends’ SM use (T1)	Disappointment	0.004	0.001–0.008
Asynchronicity (T1)	Entrapment	0.000	−0.002–0.002
Asynchronicity (T1)	Disappointment	0.000	−0.005–0.005
Availability (T1)	Entrapment	0.001	−0.002–0.005
Availability (T1)	Disappointment	−0.001	−0.006–0.005
Cue absence (T1)	Entrapment	0.000	−0.002–0.001
Cue absence (T1)	Disappointment	0.001	−0.002–0.005
Visualness (T1)	Entrapment	−0.001	−0.005–0.002
Visualness (T1)	Disappointment	0.009	0.003–0.016

Notes: ES, estimate; 95% CI, bias-corrected bootstrapped confidence interval.

### Gender differences

**Figure 3:**
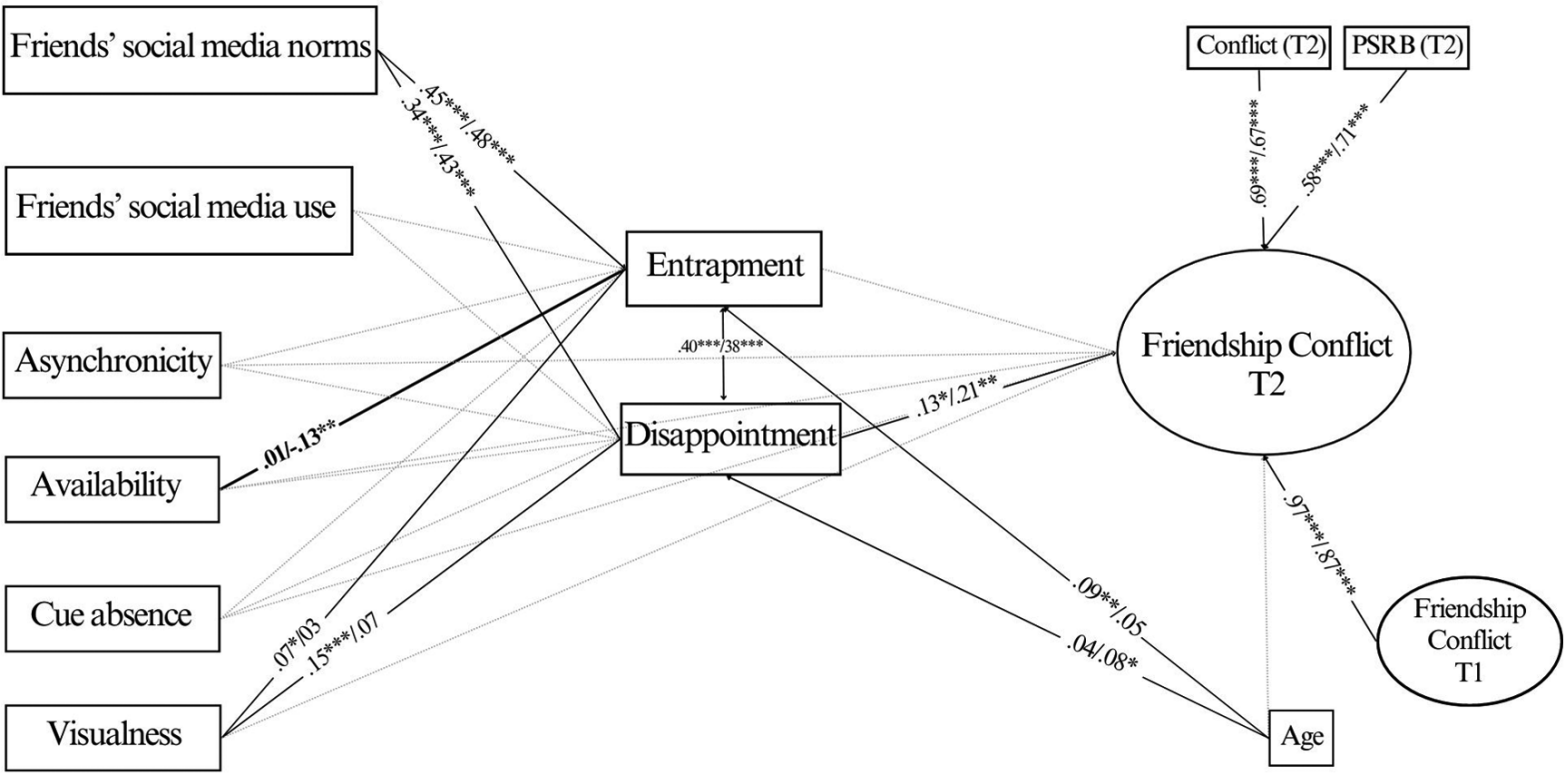
Model of the relationships between the study variables in the group of females vs. males. Notes: *N* = 1,171 (females = 703; males = 468); standardized estimates: females/males; *p *<* .05, **p *<* .01, ***p *<* .001. For sake of clarity, only relations between variables which were significant in at least one group are reported, and significantly different associations between the two groups are highlighted in bold.

Unstandardized path estimates were compared between gender groups. The overall Wald test of parameter constraints was significant (Wald χ^2^_(23)_ = 38.100, *p* = 0.025), indicating that some of the path coefficients differed across the two groups. Specifically, the association between perceived availability and entrapment was found to be negative and significant only among males (*b* = −0.10, *SE* = 0.04, *p* = 0.005, vs. *b* = 0.02, *SE* = 0.04, *p* *=* *0*.695*,* χ^2^_(1)_ = 4.786, *p* = 0.029). With regard to the other associations, we did not find any other significant differences between the two groups. However, visualness was found to be significantly associated with both entrapment and disappointment only among females. Results from the multi-group analysis are reported in Figure 3.

## Discussion

Social interactions through social media facilitate adolescents' connections with other users, especially friends, in a way that can be both beneficial [e.g., (2, 71)] and detrimental for perceived relational quality (9, 42). For example, new potential stressors in friendships are related to constant availability and accessibility to others via social media, in a way that would not be possible in the offline context (1). Specifically, adolescents may perceive the pressure to be constantly available to friends on social media and also ask them to be always responsive as well (7). When such expectations and requests on social media are not met, potential stress may arise (29). This phenomenon has been defined as “digital stress” (42), and recent literature has begun to investigate how it could affect individual and relational well-being during adolescence [e.g., (8, 9)]. Although the literature on digital stress is consistent, few studies have focused specifically on conflictual interactions among friends, in association with digital stress (1). Therefore, this study aimed to contribute to filling this gap by longitudinally testing the role of adolescents' social media expectations related to friends' norms about social media use, friends' social media use, and perceived features of the social media context in explaining different aspects digital stress and, in turn, friendship conflict over a short period of time. Moreover, differences in the studied associations between gender groups were explored. Overall, the results confirmed that both injunctive and descriptive norms and specific social media features are directly associated with digital stress and, indirectly, with friendship conflict, especially via the experience of disappointment on social media.

### Association between perceived norms and digital stress

Regarding our first hypothesis, as expected, results of the SEM analysis showed that both friends' social media norms and friends' social media use were positively associated with the two dimensions of digital stress. That is, the more adolescents perceive that using social media is a valued and expected behavior within their friends' group (i.e., injunctive norm), and that friends are used to frequently interact with, and react to their content on social media (i.e., descriptive norm), the greater the level of social media entrapment and disappointment they experience. Thus, internalizing perceived norms about social media use contribute to create expectations about how to behave with friends online; this can lead to a heightened sense of pressure to engage with social media more frequently, respond quickly to messages, or maintain a constant online presence (i.e., entrapment) and heightened emotional distress resulting from not finding friends as readily available and responsive (i.e., disappointment). However, stronger effects were found in the associations of friends' social media norms with digital stress, indicating that injunctive norms about social media use - that is, participants' perception of the use of social media as a valuable behavior by friends - are more relevant for adolescents' availability-related stress experiences, than descriptive norms about social media use – that is, the way friends are perceived to behave on social media. Therefore, adolescents may be more influenced by their perception of what is considered socially acceptable and encouraged within their friends' group than by the specific behaviors they observe on social media. Such results align with recent findings from both empirical studies and meta-analyses (22, 72), suggesting that the two types of norms have different influential impact, with injunctive norms being more effective in changing behaviors. Further, as both entrapment and disappointment may arise from individuals' motivation for affiliation, the influence of injunctive norms is reasonably more relevant for adolescents (20, 21).

However, it is also possible that adolescents' perceptions of their friends' use of social media is under- or overestimated compared to what is the reality, thereby social influence processes may be altered when occurs online (see also blinded for review). As a result, adolescents might feel pressured to conform to perceived friends' social media norms, even if their friends' actual behavior on social media does not necessarily align with these beliefs. Thus, more research is needed to better distinguish the role of friends' social media norms and friends' social media use in adolescents' online experiences.

### Association between perceived norms and friendship conflict via digital stress

Regarding our second hypothesis, indirect associations between perceived norms and friendship conflict via digital stress were found. However, contrary to what was expected, only social media disappointment, and not social media entrapment, mediated the association with conflict levels reported six months later. Although previous studies [e.g., (1)] have described entrapment as a potential source of conflict with friends, when studied together with other dimensions of digital stress (e.g., disappointment), it may have a weaker impact on relational quality. Thus, these findings suggest that the emotional distress experienced during online interactions, due to unmet expectations of their friends’ availability, is more relevant to explain increased friendship conflict than the feeling of being forced to be always available online. A possible explanation is that the pressure that stems from meeting friends' expectations is perceived as a more “normalized” behavior within peer relationships, and it might not necessarily increase interpersonal tension; as pointed out by Fox et al. (1), social media entrapment is a widespread experience that may not be detrimental *per se*, and its association with friendship conflict may depend on the balance between social benefits and costs in the relationship (73, 74). On the other hand, perceiving that friends are not adhering to shared norms and expectations may be perceived by adolescents as more serious and damaging to the relationship (29); moreover, social media disappointment is directly linked to negative emotions such as feeling sad, upset, and angry, which are more likely to spill over into conflictual relationships (52). Similarly, compared to the experience of entrapment, which is related to bad feelings over one's own failure in meeting friends' expectations (44), feelings of anger resulting from disappointment are directed to friends' failure to meet participants' demands (29). Finally, it is also likely that adolescents tend to report higher levels of conflict when they come from others' failures (7).

Overall, these results underlie the importance of understanding the mechanisms of perceptions of social norms (especially injunctive norms) in the social media environment, as they can significantly impact adolescents' experiences with friends.

### Association between perceived social media features and digital stress

With regard to the role of the perceived social media features, our third hypothesis posited that the perceived presence of asynchronicity, availability, cue absence, and visualness on social media would contribute to explaining the experiences of digital stress. However, contrary to what was expected, our findings showed that only visualness was significantly associated with disappointment. A possible explanation is that the visual nature of content on social media, whether images or videos, might evoke stronger emotional responses (2), potentially increasing the feeling of disappointment; for example, differently from offline experiences, social media visualness allows for increased adolescents’ exposure to friends' experiences, captured and shared through images and videos (4, 5, 13), thus increasing opportunities for awareness of friends' unavailability and the related disappointment of unmet demands and expectations. Furthermore, adolescents' reliance on visual content to communicate may increase expectations that friends will be responsive and give feedback, thus increasing anger when this does not occur (2).

Regarding the lack of significant associations of the other social media features with entrapment and disappointment suggests the need for a more nuanced understanding of how these features interact with adolescents' emotional responses. It is possible that additional variables, not considered in this study, play a role in shaping adolescents' perceptions and experiences associated with specific social media features.

### Association between perceived social media features and friendship conflict via digital stress

Finally, regarding our fourth hypothesis, our results indicated that the experience of social media disappointment mediated the association between perceived visuality on social media and increased friendship conflict six months later. In other words, when friends are unavailable on social media, even if their online activity (e.g., sharing visual content) reveals that they are, it may trigger adolescents' feelings of upset and disappointment, who consequently get angry and argue with their friends. Thus, visualness seems to play a role in friendship conflict through emotional responses tied to unmet expectations of friends' online availability. However, due to the lack of previous research on these associations, further exploration is needed to understand the specific mechanisms through which visualness contributes to disappointment and, in turn, to friendship conflict. In addition, recognizing the potential for stressful online experiences arising from the visual nature of online interactions that may lead to arguments and conflicts is crucial to foster healthier friendship dynamics for adolescents.

### Gender differences

Although in the current study we did not formulate detailed a-priori hypotheses about gender differences, our findings indicated that gender does not play a major role at least in this sample. The only significant difference was observed in the relation between perceived availability and entrapment, which was negative and significant among males, but not among females; that is, males who perceive high availability of social media experience lower levels of pressure to be constantly and immediately responsive to friends, compared to females. A possible explanation refers to expectations about communication patterns across genders. For example, while females may have higher expectations for constant availability within friendships, especially when friends share the same gender identity, relational dynamics among males are characterized by greater social acceptance or encouragement to maintain a more asynchronous communication style, allowing for delayed responses (1, 44). In addition, males usually rely more on offline support systems, such as in-person interactions with friends (2, 36, 75). As a result, higher perceived availability does not necessarily translate into heightened expectations for immediate responsiveness, and, in turn, this leads to lower experience of entrapment. However, as shown in the study by Fox et al. (1), males perceiving social media entrapment are more likely to engage in friendship conflict compared to females. Thus, being less used to experiencing entrapment despite the constant availability allowed by social media may decrease the threshold of tolerance and increase the likelihood of arguing with friends when pressure to be available is experienced. No other gender differences emerged in the associations under study. However, these findings should not be considered definitive, and further research on gender differences in social media experiences in association with friendship is important.

### Limitations

Although an important novelty of this study was to investigate the longitudinal associations between social media expectations and friendship conflict, via the experience of digital stress, the first limitation relates to the relatively short interval between the first and the second wave. Therefore, the conclusions drawn from our results are limited to the period of about 6 months, and future research should try to follow adolescents for a longer period of time. Second, self-report scales were used to assess all the variables in the study, and in addition, we requested participants to report their friends' social media use. To address certain shortcomings of self-reports (e.g., social desirability biases and over or under estimation of others' social media use), future research should also use objective metrics, such as having participants install an application that monitors their and their friends' actual activities on social media. Furthermore, regarding social norms, we adopted Cialdini's distinction between injunctive and descriptive norms, which typically refer to the same concept; however, in this study the two types of norms referred to slighlty different behaviors, that is, the extent of social media use is perceived as being expected (i.e., injunctive), and other-oriented social media activities, such as posting a picture of friends (i.e., descriptive). Thus, future studies should account for a better distinction of social norms, by focusing on a specific component of social media use. In addition, future research should consider possible confounding variables related to the season when data are collected (e.g., the level of other outside stimulations or after-school activities) that might affect the results. Finally, beyond gender, investigating the moderating role of other factors, such as adolescents' social and emotional skills, could provide a more comprehensive understanding of adolescents' online experiences of digital stress and their implications for friendship conflicts. In this regard, it should also be noted that, according to the concept of transformation of peer experiences (3–5), the presence of the social media features may also amplify peer influence processes (15). Indeed, it is likely that the perception of some features interacts with norms. Therefore, future studies should try to test the role of social media features as moderator in the association between norms and digital stress. Finally, as conflict is something relational, beyond the feeling of disappointment as it was conceptualized in the current study, that is, participants disappointed due to unmet friends' expectations of availability, it would also be interesting to investigate participants' perception of causing feelings of disappointment to friends, when they do not meet their expectations.

### Implications

Despite these limitations, findings from this study have several implications both from a theoretical and intervention perspective. First, this study adds to the literature on friendship by allowing a better understanding of the role of social media in adolescents' relationships with their friends. Specifically, conflictual interactions in friendship, as an indicator of low perceived friendship quality, have rarely been investigated so far, especially in association with stressful online experiences. By focusing on the distinction between different contextual factors that may contribute to forming expectations about interactions with friends on social media, this study highlighted the role of perceived norms and social media functioning in explaining the experiences of digital stress, which may turn out to worsen their friendship. Despite the influence that social media may have in transforming adolescents' peer relationships and experiences (3–5), no studies have investigated whether normative online processes and the presence of unique social media features - at least as far as they are perceived by adolescents as relevant - may represent a potential source of negative online experiences with friends. In addition, there is little, and mostly qualitative, research on the association of digital stress and conflictual interaction with friends. Thus, findings from this study suggest the introduction of new potential stressors related to the perceived context of social media and provide a better understanding of the psychological mechanisms underlying the dynamics of friendship conflicts. Furthermore, another important contribution to the literature concerns the acknowledgement of the two facets of digital stress (i.e., entrapment and disappointment), as representative of the reciprocal nature of friendship.

Second, findings from this study may have practical implications for educational programs targeting adolescents. Specifically, such programs can focus on the promotion of relational well-being, and on the prevention of negative effects related to social media use. Given that social media may contribute to increased experiences of digital stress and, consequently, levels of conflict with friends, strategies are needed to manage and cope with this stress. On the other hand, while conflict can be challenging, especially during adolescence, it also provides an opportunity to develop important problem-solving and conflict resolution skills. Therefore, these findings can inform schools and educators in implementing educational programs and interventions aimed at providing resources to help adolescents mitigate the negative impact of certain experiences on social media on the quality of their friendships. For example, strategies for effective communication during online interactions and the development of healthy social media norms should be encouraged, promoting skills and discussions on appropriate expectations for availability and emotional communication that enable adolescents to have positive social media experiences within their friendships. Furthermore, as digital stress is assumed to arise as practices of use exceed coping strategies, these findings can inform the development of tailored interventions. Most social media, in fact, are designed to facilitate social connections, and adolescents should be made aware of this potential to reap the relational benefits of their use.

## Conclusion

This study contributes to the expanding body of literature about social media experiences within friendship during adolescence. While social media can support friends' social interactions, it can also create unprecedented norms and expectations for permanent availability, thus introducing new stressors for relational quality. Specifically, our findings suggest that friends' norms about social media use and unique features of social media, especially visualness and availability, are associated with friendship conflict via increased experiences of digital stress, over a short period of time. Moreover, adolescents' emotional responses to unmet expectations on social media by friends (i.e., disappointment) was more relevant in explaining conflictual interactions, compared to their perceived pressure to satisfy friends' demands (i.e., entrapment). In conclusion, the current study supports the importance of considering social media as a context to study the relational dynamics of friendships, as this knowledge can contribute to supporting adolescents as they navigate the complexities of online experiences.

## Data Availability

The raw data supporting the conclusions of this article will be made available by the authors, without undue reservation.
